# A RecQ Protein-like 5 Haplotype is Associated With Colon Cancer

**DOI:** 10.4021/gr2010.06.214w

**Published:** 2010-05-20

**Authors:** Heather M. Ochs-Balcom, Cheryl L.Thompson, Sarah Plummer, Guangbin Luo, Thomas C. Tucker, Graham Casey, Li Li

**Affiliations:** a Department of Social and Preventive Medicine, State University of New York at Buffalo, Buffalo, New York, 14214, USA; b Department of Epidemiology and Biostatistics, Case Western Reserve University, Cleveland, Ohio, 44106, USA; c Department of Family Medicine, Case Western Reserve University/University Hospitals Case Medical Center, Cleveland, Ohio, 44106, USA; d Case Center for Transdisciplinary Research on Energetics and Cancer, Case Comprehensive Cancer Center, Case Western Reserve University, Cleveland, Ohio, 44106, USA; e Department of Preventive Medicine, University of Southern California, Los Angeles, California, 90089, USA; f Department of Genetics, Case Western Reserve University, 44106, Cleveland, Ohio, USA; g Cancer Control Program, University of Kentucky, Lexington, Kentucky, 40536, USA

**Keywords:** *RECQL5*, Colon cancer, Haplotype, Single Nucleotide Polymorphism

## Abstract

**Background:**

Emerging murine model data suggests RecQ protein-like 5 (*RECQL5*) is a tumor suppressor gene. The goal of our study was to test whether *RECQL5* gene variants are associated with colon cancer susceptibility.

**Methods:**

We examined the association of two haplotype-tagging SNPs in *RECQL5* and colon cancer in a population-based study of 390 colon cancer cases and 464 population controls.

**Results:**

While both crude and covariate-adjusted single SNP analyses were only suggestive for an association with borderline significance (p = 0.07), haplotype analysis shows that individuals carrying the T-G haplotype (rs820196 common allele and rs4789223 minor allele) were at significantly increased risk for colon cancer (OR = 1.34, 95% CI = 1.02-1.76, p = 0.05). Adjustment for age, sex, body mass index, non-steroidal anti-inflammatory use and family history of colon cancer did not alter the results.

**Conclusions:**

These results suggest that a haplotype harboring the minor allele of rs4789223 is associated with colon cancer risk. Further study of *RECQL5* as a colon cancer susceptibility gene is warranted, particularly with respect to variants in linkage disequilibrium with rs4789223.

## Introduction

*RECQL5* is a member of the RecQ helicase family which includes a number of disease-causing genes implicated in several cancer prone syndrome such as Bloom syndrome, Werner syndrome, and Rothmund-Thomson syndrome [1]. Recent work in Apc^min/+^ mice harboring Recql5 deficiency (Recql5^-/-^) showed marked increase in microadenoma formation compared to Recql5^+/+^ mice [2]. Another recent study showed that mice deficient for *Recql5*, the mouse homologue of human *RECQL5* are predisposed to a wide variety of other sporadic cancers, establishing this protein as an important tumor suppressor in mice [3]. This evidence along with the fact that mouse *Recql5* and human *RECQL5* are highly conserved [3], suggests human *RECQL5* may also have an important tumor suppressing function and therefore certain genetic variants that affect the function of this *RECQL5* may then alter the risk of cancer in humans. In this study, we tested the hypothesis that *RECQL5* variants are associated with colon cancer in a population-based case control study.

## Patients and Methods

### Study population

The study design and methodology for this population-based case-control study has been described elsewhere, and all human subjects approvals were obtained [4]. Briefly, eligible cases were identified through the population-based Kentucky Cancer Registry which is part of both the National Cancer Institutes' Surveillance, Epidemiology and End Results (SEER) program and the Centers for Disease Control and Preventions' National Program of Cancer Registries (NPCR). Residents living in Kentucky at the time of diagnosis between 2003 and 2006 were included in this study. We identified all incident and primary colon cancer cases reported within 6 months of diagnosis. We used random digital dialing to recruit controls. The area codes and exchanges of the cases were used as a proxy for frequency match of the residential locations of the cases, along with randomly-generated 4 digit numbers for recruitment of cancer-free controls. We collected information on demographics, family history of colorectal cancer, and personal cancer history via telephone questionnaire. Each participant received a phlebotomy kit with instructions for fasting blood sample collection, consent forms, and a self-administered lifestyle risk factor questionnaire (RFQ) (http://epi.grants.cancer.gov/documents/CFR/center_questionnaires/Colon/LA/ColonRiskFactor_USC.pdf) developed by the National Cancer Institute Colon Cancer Familial Cancer Registry to collect detailed information on family history of colorectal cancer, lifestyle and behavioral risk factors. Individuals with known inflammatory bowel diseases, family history of familial adenomatous polyposis, and hereditary non-polyposis colorectal cancer were excluded from the study, and controls were required to be at least 30 years of age. All participants provided written informed consent. The study was approved by the Institutional Review Boards of the University of Kentucky, Lexington, and Case Western Reserve University/University Hospitals of Cleveland. The participation rates were 72.2% for the cases, and 62.5% for eligible controls.

### SNP selection and genotyping

Common genetic variation in the *RECQL5* gene was identified by querying the Genome Variation Server (http://gvs.gs.washington.edu/GVS) for SNPs within *RECQL5* and 5 kb upstream and downstream with a minor allele frequency (MAF) of at least 5%. Haplotype tagging SNPs were selected based on MAF and linkage disequilibrium (LD) patterns in the HapMap Caucasian samples with preference given to non-synonymous coding variants. Caucasian samples were used as a reference as over 90% of our sample self-report as Caucasian. A single non-synonymous coding SNP was identified (rs820196) in this region. No other non-synonymous coding or regulatory SNPs were identified. The addition of one more SNP (rs4789223) was identified to sufficiently tag the entire region with 85% coverage.

Blood samples were shipped overnight on frozen ice pack. Upon receipt, samples were spun for 15 minutes at 600 x g and aliquots of plasma and concentrated buffy coat were prepared and frozen at ^-^80°C. Genomic DNA was extracted from thawed buffy coat fractions using the Qiagen EZ1 Biorobot. Genotypes were determined using 1.25 ng of genomic DNA with predesigned Taqman assays from Applied Biosystems (C_7978409_10, and C_7978435_20) and RealMasterMixProbe +ROX (5 Prime). Assays were read using the 7900HT Real-Time PCR system (Applied Biosystems). Two percent of samples were repeated with a concordance of 100%. Neither SNP deviated significantly from Hardy-Weinberg proportions.

### Statistical analysis

We limited our analyses to Caucasians only due to the few individuals in other racial/ethnic groups (N = 61). Of 397 cases and 467 controls that completed the study, seven cases and three controls were excluded due to missing SNP data. The final set for analysis includes 390 cases and 464 controls.

We examined differences in demographic and risk factors using chi-square tests and student’s t-tests. We estimated allele and genotype frequencies and assessed deviations from Hardy-Weinberg equilibrium in cases and controls separately using chi-square tests with 1 degree of freedom. We computed odds ratios (ORs) and 95% confidence intervals using unconditional logistic regression models under log-additive genetic models for each of SNP. We estimated both crude ORs and ORs adjusted for age, sex, non-steroidal anti-inflammatory drug (NSAID) use, body mass index (BMI), and any family history of colorectal cancer. NSAID use was defined as self-reported intake of aspirin or ibuprofen at least twice weekly for a period of six months or longer.

We estimated haplotype frequencies of 2-SNP haplotypes using the expectation maximization algorithm within the UNPHASED software, and estimated risk associated with each haplotype with the most common haplotype as the reference category using unconditional logistic regression [5, 6].

## Results

We selected two tagSNPs to represent genetic variation at the *RECQL5* locus. The rs820196 SNP is located in exon 1 (71139134 in build 36) and is a nonsynonymous mutation that results in an amino acid change from aspartic acid to glycine. The rs4789223 SNP is intronic.

Table 1 shows that similar to previous reports on this sample [4], cases were more likely have a higher BMI (p = 0.001) and a family history of colorectal cancer (p = 0.001). Cases were also slightly older at diagnosis than the controls at time of recruitment; the mean age was 63.0 and 58.1 years for cases and controls, respectively. The cases comprised a higher proportion of males than the controls (p = 0.001).

**Table 1:** Kentucky Colon Cancer Study Demographics

Variables, Mean (SD) or n (%)	Cases (n = 390)	Controls (n = 464)	p-value^a^
Age, yr	63.0 (10.5)	58.1 (11.1)	0.001
Male	196 (50)	170 (37)	
Female	194 (50)	294 (63)	0.001
Regular NSAID use^b^	217 (64)	290 (70)	0.08
Family history of colorectal cancer	86 (25)	62 (15)	0.001
Body mass index (kg/m^2^)	29.2 (6.3)	27.9 (5.8)	0.002

a Student’s t-test or chi-square for differences, complete data on covariates available for 340 cases and 415 controls.

b Use at least twice per week ≥ 6 months.

Table 2 shows the allele and genotype frequencies for *RECQL5* SNPs. The minor allele frequency for rs820196 in our control sample (0.23) was slightly higher than the HapMap CEPH data (0.21). The MAF for rs4789223 was very similar; our control frequency was 0.39 and Hapmap was 0.38. Allelic association tests were not significant for rs4789223 (p = 0.07) or rs820196 (p = 0.60). Table 2 shows the adjusted OR of 1.21 (95% CI = 0.98 -1.49).

**Table 2:** Allele and Genotype Frequencies and Association Tests for *RECQL5* SNPs

SNP	Total	Genotype			MAF	Allele χ^2^, p-value	Crude OR (95% CI)	Adjusted OR^a^ (95% CI)
rs820196		TT	TC	CC	C			
Cases	390	0.58	0.36	0.06	0.24	0.27, 0.60	1.06 (0.85, 1.32)	1.05 (0.83, 1.34)
Controls	464	0.60	0.34	0.06	0.23			
rs4789223		AA	AG	GG	G			
Cases	390	0.34	0.45	0.21	0.43	3.42, 0.07	1.19 (0.98, 1.43)	1.21 (0.98, 1.49)
Controls	464	0.38	0.46	0.16	0.39			

a Per allele odds ratios adjusted for age, sex, BMI, NSAID use and family history of colorectal cancer.

The haplotype results in Table 3 suggest that individuals who carry the T-G haplotype, that is the common allele of rs820196 and the minor allele of rs4709223, are at increased risk compared to individuals who carry the common alleles together on a haplotype, crude OR: 1.34, 95% CI = 1.02 - 1.76. The T-G haplotype was more common in cases; approximately 20% of the cases and 16% of the controls carried this haplotype. Adjustment for covariates only strengthened the estimates.

**Table 3:** *RECQL5* Colon Cancer Haplotype Association Tests

rs820196-rs4789223 haplotype^a^	Case frequency	Control frequency	OR (95% CI), p-value	Adjusted OR (95% CI),^b^ p-value
T-A (common alleles)	0.57	0.61	1.0 (reference)	1.0 (reference)
C-G (2 minor alleles)	0.23	0.23	1.13 (0.83, 1.37), 0.62	1.13 (0.87, 1.47), 0.72
T-G	0.20	0.16	1.34 (1.02, 1.76), 0.05	1.39 (1.04, 1.87), 0.04

a C-A haplotype too rare to consider; frequency of 0.001 in cases, absent in controls.

b Adjusted for age, sex, BMI, NSAID use and family history of colorectal cancer.

## Discussion

Colon cancer is a complex disease that is in part defined by the involvement of multiple genes with small effects influencing susceptibility. The search for susceptibility genes for colon cancer remains an active area of research in the form of both genome-wide association studies (GWAS) and candidate gene studies, each of which have their respective strengths and limitations for gene discovery. Many independent associations have been identified using the genome-wide approach, and these regions do not include the region on chr17 harboring *RECQL5* [7]. Nonetheless, many other important candidate genes are likely to remain undiscovered using the GWAS approach, particularly because the large number of statistical tests are prohibitive to examining more than single SNP models (i.e. multi-allelic or haplotype models) at the first stage analysis. We hypothesized that *RECQL5* is a novel candidate gene for colon cancer given the known involvement in DNA repair and homologous recombination and association of other members of this family with known cancer disorders. Our tag-SNP association analysis did not reveal evidence for an association with colon cancer and *RECQL5*. We did however find a significant association with a haplotype harboring the minor allele of rs4789223. These results are not surprising in a complex disease such as colon cancer, where multiple genes may have small effects.

To date, there are no known diseases or syndromes associated with variants or mutations in *RECQL5*. First cloned in silico in 1998, *RECQL5* was found to be expressed in most tissues, especially pancreas and testis, unlike other members of the family whose expression appears to be more tissue-specific [8]. *RECQL5* is known to be alternatively spliced to produce three different isoforms, *RECQL5α*, *RECQL5β*, and *RECQL5γ* [9, 10].

The rs820196 SNP in *RECQL5* is a nonsynonymous coding SNP in exon 9, which results in a change from aspartic acid to glycine; rs4789223, significantly associated with increased colon cancer risk in the haplotype analysis, is mapped at intron 8 and is not coding. There is moderate LD between these 2 SNPs; r-squared is 0.44.

Our study had 80% power to detect ORs ranging from approximately 1.34 to 1.40 given minor allele frequencies between 0.43 and 0.23, respectively, and thus this could explain the lack of definitive evidence of single SNP significance. Since haplotype analyses have better power to detect association when LD is taken into consideration, this may explain why the haplotype analyses were significant but not the SNPs alone [11]. If these results are replicated, the haplotype may harbor an untyped variant that could affect susceptibility.

In summary, we found that a haplotype in *RECQL5 tagged by* rs4789223 or other variants in LD with this SNP is associated with colon cancer in a case control study. Our results in combination with emerging evidence from model systems support *RECQL5* as a novel susceptibility for colon cancer, and are worthy of further replication.
